# Human Papillomavirus–Induced Cancer: Late Relapse in a Patient Treated With Tumor Necrosis Factor-Alpha Inhibitor

**DOI:** 10.1200/JGO.2016.005835

**Published:** 2016-08-24

**Authors:** Gabriel Madeira Werberich, Tatiana Strava, Carolina Vizioli, Gustavo dos Santos Fernandes

**Affiliations:** All authors: Hospital Sírio-Libanês, Brasília, Brazil.

## CASE REPORT

In 1989, a 48-year-old white woman with well-controlled hypertension and type 2 diabetes was treated with abdominoperineal resection with definitive colostomy because of a 4-cm anal squamous cell cancerous tumor, with zero of four lymph nodes affected. The patient received adjuvant radiotherapy with 5,000 cGy in 25 sessions. After 1 year, she developed hepatitis C and was treated with peginterferon, with good disease control at that time. However, in 2009, at age 68 years, she developed psoriatic arthritis. She began taking etanercept in 2013 with clinical benefit and continued taking the drug until recently.

**Fig 1:**
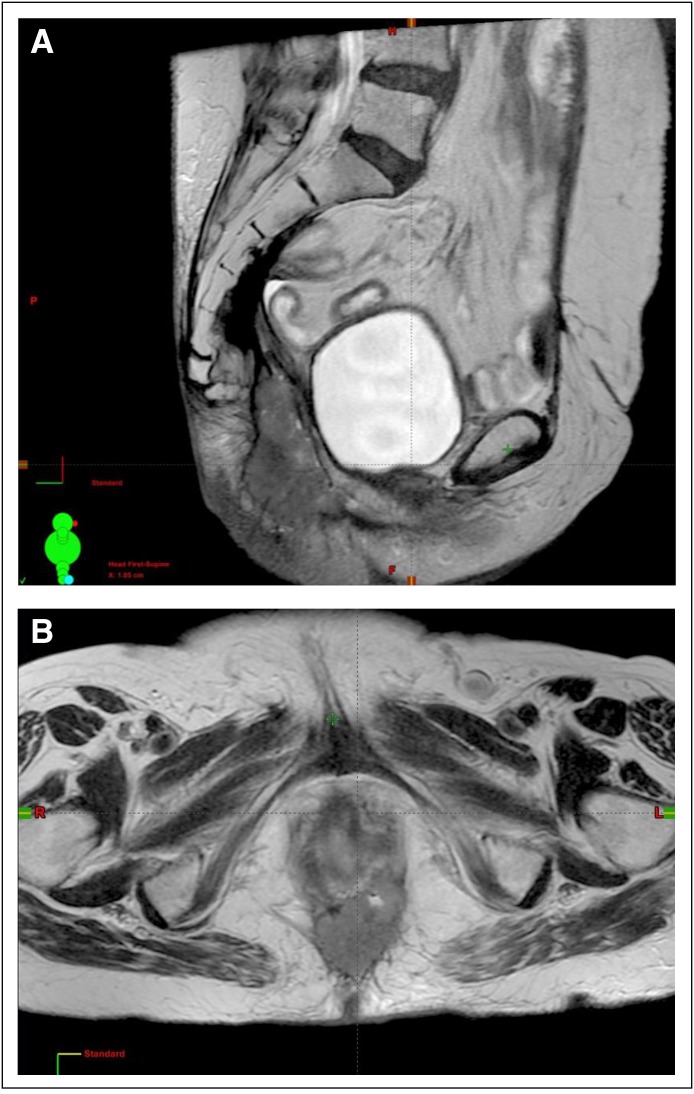
(A) Sagittal T2-weighted magnetic resonance image of the pelvis showing expansive 5.6 cm lesion at rectal topography. (B) Axial T2-weighted magnetic resonance image of the pelvis showing expansive lesion at rectal topography.

**Fig 2:**
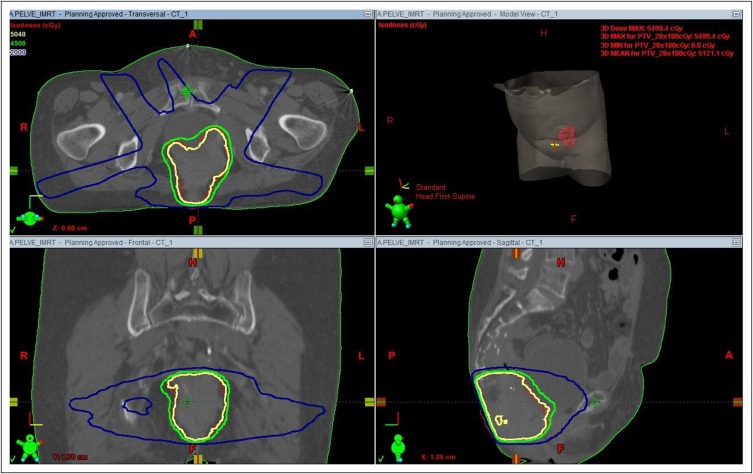
Radiotherapy treatment plan.

She presented to our clinic with hypogastrium pain in August 2015. A magnetic resonance imaging scan of the pelvis showed an expansive 5.6-cm lesion in rectal topography (Fig 1A and 1B). The [^18^F]fluorodeoxyglucose positron emission tomography/computed tomography scan showed a hypermetabolic lesion in the same rectal topography and regional lymph nodes, with a small increase in metabolism. Metastatic disease was ruled out. Gynecologic examination showed no lesions suggestive of primary gynecologic cancer. The patient’s carcinoembryonic antigen level was 11.8 ng/mL. An ultrasound-guided biopsy of the lesion in the rectal region showed squamous cell carcinoma; immunohistochemistry results were p16-positive, cytokeratin 5-positive, cytokeratin 7-positive, cytokeratin 20-negative, and p63-positive. The treatment plan was concomitant chemoradiotherapy (Fig 2) with fluorouracil and mitomycin (Nigro protocol).

## DISCUSSION

Tumor necrosis factor (TNF)–alpha is a potent proinflammatory cytokine involved in the pathogenesis of many inflammatory and autoimmune conditions, including psoriatic arthritis. The development of TNF inhibitors was an important advance in the treatment of these diseases, although their use remains controversial in patients with a history of cancer because of the possible effect of this cytokine on carcinogenesis and tumor progression.^1^

Showing a true association between TNF blockade and the development of cancer is difficult for many reasons, such as event underreporting; the risk from underlying disease (for example, patients with rheumatoid arthritis have a greater risk of developing some malignancies, such as lymphoma^2-4^); and the use of immunosuppressive medications (methotrexate, azathioprine, cyclophosphamide). Also, patients with cancer are excluded from clinical trials with TNF inhibitors because of a concern over a potentially increased risk of developing cancer. Concerns about a possible correlation between TNF blockade and cancer first arose from postmarketing reports of 26 cases of lymphoma among patients treated with etanercept and infliximab.^5^

A meta-analysis including 3,493 patients with rheumatoid arthritis who received TNF inhibitors (infliximab and adalimumab) and 1,512 patients who received placebo showed more malignancies (0.8%) in the TNF-inhibitor group compared with those who received placebo (0.2%), with a pooled odds ratio of 3.3 (95% CI, 1.2 to 9.1). Risks appeared to be dose dependent, with patients receiving high-dose therapy having the greatest risk (odds ratio, 4.3; 95% CI, 1.6 to 11.8).^6^ A 180-patient trial of etanercept for antineutrophil cytoplasmic antibody–associated granulomatous vasculitis revealed that six solid cancers developed in the etanercept group compared with none among the placebo controls (*P* = .01).^7^

Recently, an observational prospective trial using the data from the British Society for Rheumatology Biologics Register, from 2001 to 2011, showed that TNF inhibitors did not increase the solid tumor risk in this population. The weakness of this study was selection bias: patients with a previous cancer diagnosis were not included, meaning that the results cannot be extrapolated to this type of patient, and the physicians could select patients with a low risk of cancer.^8^

A systematic review of epidemiologic studies that included 22 articles also did not demonstrate an association between TNF inhibitor use and solid tumors in a population without a previous cancer diagnosis. It is important to note that the only two anal carcinomas were observed in the TNF-inhibitor group.^9^

When it comes to human papilloma virus–induced cancers (cervical, head and neck, anal), trials show that perhaps there is a relationship between treatment-induced immunosuppression and cancer development. It is known that immunosuppression is a risk factor for virus-associated cancers, and TNF-inhibitor use decreases host defense against viral infections. A nationwide register-based cohort study in Sweden, including 9,629 patients who received TNF inhibitors between 2001 and 2012, showed a double risk of invasive cervical cancer (hazard ratio, 2.10; 95% CI, 1.04 to 4.23).^10^ There is one Japanese case report of a 34-year-old HIV-negative patient who presented with anal cancer after three sessions of infliximab administration for Crohn disease, suggested by a rising serum concentration of carcinoembryonic antigen.^11^

The patient discussed in this case report was unusual because of her late relapse, and the immune-modulating potential of her etanercept therapy may have contributed to this clinical situation. The temporal relationship between the recrudescence of her cancer after years without evidence of cancer and initiation of etanercept therapy is consistent with this idea.

In conclusion, TNF-alpha inhibitors are associated with serious adverse effects, including malignancies. However, these potential risks should be evaluated in each patient, considering disease activity and potential benefits of a targeted treatment, as well as adverse effects associated with conventional treatments, including the risk of malignancy induction, the risk of malignancy of the disease itself, and the presence of other risk factors, such as smoking, previous infection with human papillomavirus, or previous cancer.
